# Exploring the Relationship Between Parental Trauma, Parenting Style, and Children’s Medical Treatment Adherence: Cross-Sectional Study

**DOI:** 10.2196/72406

**Published:** 2026-07-21

**Authors:** Margaret Stull, Olga Vsevolozhskaya, Kristopher Collman, Rachael Steinken, Christina Rios, Amy L Meadows

**Affiliations:** 1 Department of Psychiatry, College of Medicine, University of Kentucky, 245 Fountain Court, Lexington, KY, 40509, United States, 1 5025427512; 2 Department of Biostatistics, College of Public Health, University of Kentucky, Lexington, KY, United States; 3 Department of Psychiatry, College of Medicine, The Ohio State University, Columbus, OH, United States; 4 People's Health Center and Martindale-Brightwood Health Center, Health Net, Woodland Hills, IN, United States

**Keywords:** adverse childhood experience, ACEs, parenting, adherence, trauma

## Abstract

**Background:**

Medication adherence is defined as the degree to which the medications taken reflect the prescribed intention and is influenced by various factors. Different factors, such as parental trauma or parenting style, may influence their child or children’s medical treatment adherence. Authoritative, or “flexible,” parenting is known to develop the most nurturing relationship between parent and child or children. The relationship between parental factors, such as parenting style and trauma level, and their influence on child medication adherence is being explored.

**Objective:**

This study examines the relationship between parental trauma, parenting style, and their child or children’s adherence to medical recommendations.

**Methods:**

Participants were recruited through Amazon’s Mechanical Turk, an online crowdsourcing platform. Participants were aged 18 years or older, a caregiver for at least 1 child younger than the age of 18 years, and had at least 1 child who was prescribed medication within the past 3 months. Parental trauma level was measured via the Adverse Childhood Experiences Questionnaire, and parenting style was measured via the Parental Authority Questionnaire–Revised. Descriptive statistics, including chi-square tests, 2-tailed *t* tests, and adjusted logistic regressions, were used to determine the association between parental trauma, parenting style, and their child or children’s medical adherence.

**Results:**

A total of 720 participant responses were analyzed. Children who were medically adherent were younger (*P*=.02). Caregivers’ mean age was 36.8 (SD 7.97) years, and 35.4% (n=255) identified as male. An increase in caregivers’ adverse childhood experiences score was marginally associated with an increased risk of medication nonadherence among their children (adjusted odds ratio 0.94; *P*=.07).

**Conclusions:**

Similar to other studies, this study showed that parents of children who adhere to medical treatment practice an authoritative parenting style. Uniquely, it also showed that an increased adverse childhood experiences score was only marginally associated with an increased risk of medical nonadherence.

## Introduction

Medication adherence is defined as the degree to which the medications taken reflect the prescribed intention and is influenced by various factors, including regimen complexity, personal beliefs, cognitive function, perceived quality of the clinical relationship, and familial associations [1]. Recent studies have sought to expand on the knowledge of the relationship between parental factors, such as parenting style and trauma level, and their influence on child medication adherence. It is probable that distressing events experienced by parents are passed to their child or children through differing elements via the practice of parenting. For example, multiple previous studies have shown that parental conflict is associated with poor medical adherence, specifically in glycemic control in diabetic adolescents [2]. In 1975, the “Ghosts in the Nursery” theory was generated by Selma Faiberg to explain the intergenerational transmission of trauma from parent to child [3,4]. It represents the “tendency of parents to bring to the rearing of their children the unresolved issues of their own childhoods” [4]. This phenomenon has since been observed in a variety of ecological contexts, including vulnerable, clinical, outpatient, homeless, rural, and urban populations [1,3,5,6]. These occurrences reinforce the relevance of the relationship between parental factors, such as trauma, and their child or children’s medical adherence.

Current research regarding pediatric treatment adherence, particularly in the context of children with asthma or diabetes, has focused on mediating factors similar to those discussed in prior studies regarding the intergenerational effects of parental adverse childhood experiences (ACE) exposure on children’s health [2,7-11]. ACE are defined as categories of child maltreatment and household dysfunction experienced before the age of 18 years; they are a way to conceptualize trauma [12,13]. The effects of intergenerational ACE may include parental stress, depressive symptoms, and household dysfunction and conflict [1,2,13]. The presence of certain ACE has been associated with treatment noncompliance and negative health behaviors and outcomes [14,15]. While it is generally understood that children have lower levels of treatment adherence than adults, especially prior to adolescence, these studies suggest that decreased adherence may be related to parenting stress, level of parental confidence, and/or other social and emotional factors linked to caregivers with a prior history of trauma [13,16].

At least 1 study linking parental factors, such as trauma, to decreased medical treatment adherence demonstrated that a higher level of parental distress and child or children’s behavioral problems were associated with increased medication adherence. This could possibly be due to changes in parental monitoring caused by the circumstances of raising a complex child or children, particularly if they require medications to assist in treating behavioral disturbances (including symptoms of impulsivity and/or hyperactivity) [9]. A second study found no link between maternal or paternal ACE scores and increased sick visits or delayed immunizations in children up to the age of 2 years but also found that higher maternal ACE scores were associated with decreased adherence to schedules for well-child visits [17].

Prior research has demonstrated that paternal stress level and parenting style impact children’s adherence to differing regimens [10]. Authoritarian parenting is defined as having a “one-way” mode of communication where the parent establishes strict rules and the child obeys without explanation; mistakes made by the child typically lead to punishment. Authoritative, or “flexible,” parenting allows for the development of a nurturing relationship between the child and the parent. This parenting style generally leads to the healthiest outcomes for children as it is based on family expectations. Permissive parenting is described as having a warm, nurturing relationship between parent and child or children with minimal expectations and explanations; the child or children and the parent see each other as “friends” [10,18]. Studies suggest that authoritative parenting styles that practice warmth have been associated with increased treatment adherence in adolescents with type 1 diabetes and that general positive outcomes displayed by using this style of relationship are consistent across differing gender, ethnic, and sociodemographic backgrounds [2]. This is hypothesized to be due to a reduction in conflict, an increase in cohesion, and the development of self-control, and/or self-efficacy [10,11]. Given the central role that parenting style has on child medication adherence, more research is warranted regarding how parental trauma and its effects on parenting behavior may affect medication use and treatment adherence in children. This research is especially important considering that very few studies have examined the broader role that the aforementioned relationship has on medical adherence in children. Historically, studies have only examined this relationship in instances of chronic or specific medical conditions.

The goal of this study was to characterize the relationship between parental trauma, parenting style, and their child or children’s adherence to medical recommendations. A crowdsourcing framework was used to recruit parents whose child or children had been on a medically recommended treatment in the prior 3 months. By adding to the growing body of work that examines intergenerational risk factors, this exploratory study could help to define potential points for future interventions.

## Methods

### Ethical Considerations

All study procedures were reviewed and approved by the University of Kentucky Institutional Review Board (protocol 46099) in accordance with the Declaration of Helsinki. Participants were provided information about the study in a cover letter and checked a box to indicate that they understood and consented to participate in the study. Upon completion of the screening questionnaire, participants earned US $0.05 if they did not qualify and US $3.00 if they qualified and completed the remainder of the questionnaire.

### Recruitment

Participants were recruited using the Amazon Mechanical Turk (mTurk), an online crowdsourcing platform [19,20]. Potential participants had registered on Amazon to perform Human Intelligence Tasks in return for compensation. There are 85,000 people registered on mTurk in the United States [21]. All potential participants were required to have a 99% prior task approval rate and have at least 500 completed previous tasks to be considered eligible, which demonstrates the participant’s knowledge of the platform as well as the increased effort that they have previously demonstrated toward similar tasks. Participants then completed a brief screening questionnaire to ensure eligibility, including that they were aged 18 years or older, a caregiver for at least 1 child under the age of 18 years, and their child or children had been prescribed medication within the prior 3 months. The questionnaire was estimated to take 30 minutes or less and was distributed in a closed-access mode. It asked 4 questions to confirm potential participants’ understanding of the information. Participation was voluntary, and participants were excluded if they reported that they did not have children, had a non-US IP address, or answered questions about their demographics (including age and number of children) inconsistently. It involved no procedures other than a questionnaire.

### Measures

#### Demographic Information

Demographic information was obtained via an online screening questionnaire, including the participant’s age, sex, education, race, income level, employment status, marital status, number of children, and their child or children’s age and sex.

#### Questionnaires

We evaluated the parenting style using the Parental Authority Questionnaire–Revised, developed by Reitman et al [22]. This questionnaire was revised from the Parental Authority Questionnaire, developed by Buri [23]. In the revised version, the format of the questionnaire was changed from a second-person to first-person point of view, and the reading level of the questionnaire was changed to an approximately sixth-grade level. The Parental Authority Questionnaire was originally developed for parents of children aged 3 to 8 years to self-report their beliefs about parenting their child. However, it has been used in other populations such as adolescents [24,25]. The questionnaire included three 10-item scales representing authoritative, authoritarian, and permissive parenting styles. Each item was rated based on a 5-point Likert scale (5=“Strongly Agree,” 4=“Agree,” 3=“Neither Agree Nor Disagree,” 2=“Disagree,” and 1=“Strongly Disagree”). Each subscale score ranged from 10 to 50. A higher score indicated that the participant more closely followed the indicated parenting style.

We measured participants’ ACE using the Adverse Childhood Experiences Questionnaire, developed by the Centers for Disease Control and Prevention and Kaiser Permanente in 1998 [12]. The questionnaire evaluates 3 types of ACE and/or 10 types of trauma: abuse (physical, emotional, and sexual), neglect (physical and emotional), and household dysfunction (mental illness, mother treated violently, incarcerated relative, substance abuse, and divorce). There were 10 questions, each used to evaluate 1 type of trauma mentioned earlier, beginning with the phrase, “While you were growing up, during your first 18 years of life …” The question had answer choices of either “Yes” or “No.” If the participant answered “Yes,” that question got a score of 1; subsequently, if the participant answered “No,” that question got a score of 0. The total ACE score was calculated by summing the individual scores of all 10 questions. A higher score indicated that more ACEs were experienced during the participant’s childhood.

Adherence was evaluated using 2 questions: “How often has your child taken medication as prescribed in the past 3 months?” and “How often has your child followed his or her medical treatment instructions in the past 3 months?” Each question had 5 answer choices: “Always,” “Most of the Time,” “About Half of the Time,” “Mostly Not But Once in a While,” and “Never.” If participants answered “Always” for both questions, they would be categorized into the “Adherent” group. For the purposes of this study, any other answers were dichotomized to the “Nonadherent” group. Additional information was obtained on whether the medication was for an acute or chronic condition as well as the complexity of the medical regimen.

### Statistical Approach

Descriptive statistics were used to estimate sample characteristics and adherence rates (overall and by adherence type). Chi-square tests and *t* tests were employed to statistically examine the bivariate association between predictor variables and the categorical outcomes of interest. Adjusted logistic regressions were used to evaluate the association between parental ACE and child medication treatment and use adherence. A backward stepwise logistic elimination procedure was used to identify the most parsimonious model. All *P* values were 2-sided. Analyses were performed using R statistical software (version 4.1.2; R Foundation for Statistical Computing) [26].

## Results

A total of 740 participants completed the study between February 1, 2019, and July 7, 2019, with 723 participants completing all study measures. Only completed surveys were included in the analysis component of this study. Three results were excluded from analysis because of incomplete data.

Table 1 describes characteristics of sample children (n=720); the majority of children (56.2%) were aged over 7 years, 59.3% of children were male, and 64% had some chronic condition (eg, diabetes, asthma, eczema, or attention-deficit/hyperactivity disorder). Children who were adherent to medication treatment instructions and use tended to be younger (aged younger than 13 y; *P*=.02), with medications primarily administered by their caregivers (83.2% adherent vs 71.9% nonadherent; *P*=.001), and without perceived difficulties in taking their medications (41.9% adherent vs 21.3% nonadherent; *P*<.001). There were no statistically significant differences between adherent and nonadherent children in terms of sex (60.5% male vs 57.3% nonmale), presence of a chronic condition (64% in both presence and absence of a chronic condition), the length of medication prescription (57.2% of children taking a prescription for over 4 wk vs 48.7% of children not taking prescription for over 4 wk), the average number of prescriptions (1.62 vs 1.64 prescriptions), or the frequency of medication use (49.2% daily use vs 44.9% nondaily use). All of these measures had a *P*>.05.

**Table 1.** Characteristics of adherent and nonadherent children (N=720)^a^.

Child-level characteristic	Nonadherent (n=267), n (%)	Adherent (n=453), n (%)	Total (n=720), n (%)	*P* value
Male	153 (57.3)	274 (60.5)	427 (59.3)	.32
Age (y)				.02
<1	5 (1.9)	18 (4)	22 (3.2)	
1-3	54 (20.2)	66 (14.6)	120 (16.7)	
4-6	56 (21)	116 (25.6)	172 (23.9)	
7-9	43 (16.1)	96 (21.2)	139 (19.3)	
10-12	42 (15.7)	80 (17.7)	122 (16.9)	
13-15	35 (13.1)	44 (9.7)	79 (11)	
16-18	32 (12)	33 (7.3)	65 (9)	
Chronic condition^b^	171 (64)	290 (64)	461 (64)	.99
Length of prescription				.07
Less than 3 days	10 (3.7)	10 (2.2)	20 (2.8)	
More than 3 days, but less than 1 week	40 (15)	62 (13.7)	102 (14.2)	
More than 1 week, but less than 2 weeks	53 (19.9)	88 (19.4)	141 (19.6)	
Between 2 and 4 weeks	34 (12.7)	34 (7.5)	68 (9.4)	
More than 4 weeks	130 (48.7)	259 (57.2)	389 (54)	
Number of prescriptions	1.68 (0.84)	1.62 (0.87)	1.64 (0.86)	.41
Frequency of use				.34
Less than once daily	5 (1.9)	3 (0.7)	8 (1.1)	
Daily	120 (44.9)	223 (49.2)	343 (47.6)	
Twice daily	106 (39.7)	177 (39.1)	283 (39.3)	
3 times daily	22 (8.2)	23 (5.1)	45 (6.3)	
4 or more times daily	5 (1.9)	10 (2.2)	15 (2.1)	
As needed or PRN^c^	9 (3.4)	17 (3.8)	26 (3.6)	
Difficulty in use^d^				<.001
Extremely easy	57 (21.3)	190 (41.9)	247 (34.3)	
Somewhat easy	126 (47.2)	178 (39.3)	304 (42.2)	
Neither easy nor difficult	30 (11.2)	28 (6.2)	58 (8.1)	
Somewhat difficult	45 (16.9)	52 (11.5)	97 (13.5)	
Extremely difficult	9 (3.4)	5 (1.1)	14 (1.9)	
Who administers medication?				.001
Child	32 (12)	34 (7.5)	66 (9.2)	
Caregiver	192 (71.9)	377 (83.2)	569 (79)	
Child and Caregiver	43 (16.1)	42 (9.3)	85 (11.8)	

a Adherence was defined as both always following medication treatment instructions in the past 3 months and always taking medications as prescribed in the last 3 months.

b Examples include diabetes, asthma, eczema, and attention-deficit/hyperactivity disorder.

c PRN: pro re nata.

d Perceived level of difficulty in taking medication.

Table 2 describes characteristics of sample caregivers (n=720); their mean age was 36.8 years and 35.4% identified as male. Caregivers of children who were adherent to medication treatment instructions and use tended to be White (86.3% adherent versus 77.9% nonadherent; *P*=.002) and had an authoritative parenting style (authoritative score of 41.2 for adherent vs authoritative score of 40.3 for nonadherent; *P*<.001). There were no statistically significant differences between caregivers of adherent and nonadherent children in terms of level of education (received a bachelor’s degree or higher; 56.7% for adherent vs 59.2% for nonadherent), employment status (employed; 74.6% for adherent vs 74.9% for nonadherent), marital status (single; 19.6% for adherent vs 19.9% for nonadherent), the total number of children (had 2 children; 75.5% for adherent vs 69.7% for nonadherent), substance use in the past 3 months, or the average number of ACE (2.67, SD 2.62 ACE for adherent vs 2.97, SD 2.76 ACE for nonadherent; *P*=.15).

**Table 2.** Characteristics of caregivers by children’s medication adherence status (n=720).

Caregiver-level characteristic	Nonadherent (n=267)	Adherent (n=453)	Total (n=720)	*P* value
Male	90 (33.7)	165 (36.4)	255 (35.4)	.51
Age (y), mean (SD)	36.8 (8.13)	36.8 (7.88)	36.8 (7.97)	.94
Race				.002
White	208 (77.9)	391 (86.3)	599 (83.2)	
Black or African American	18 (6.7)	29 (6.4)	47 (6.5)	
Other	41 (15.4)	33 (7.3)	74 (10.3)	
Level of education				.06
High school	9 (3.4)	35 (7.7)	44 (6.1)	
Some college	100 (37.5)	161 (35.5)	261 (36.3)	
Bachelor’s degree or higher	158 (59.2)	257 (56.7)	415 (57.6)	
Employed	200 (74.9)	338 (74.6)	538 (74.7)	.99
Single	53 (19.9)	89 (19.6)	142 (19.7)	.93
Total number of children				.37
1	90 (33.7)	180 (39.7)	270 (37.5)	
2	96 (36)	162 (35.8)	258 (35.8)	
3	55 (20.6)	70 (15.5)	125 (17.4)	
4+	25 (9.4)	39 (8.6)	64 (8.9)	
Parenting style, mean (SD)				
Permissive score	25.2 (5.43)	24.6 (5.65)	24.8 (5.57)	.14
Authoritative score	40.3 (4.42)	41.2 (4.30)	40.9 (4.36)	<.001
Authoritarian score	32.3 (5.42)	32.7 (5.88)	32.5 (5.71)	.41
Substance use^b^				
Alcohol	172 (64.4)	288 (63.6)	460 (63.9)	.88
Cigarette	92 (34.5)	129 (28.5)	221 (30.7)	.11
Marijuana	60 (22.5)	86 (19)	146 (20.3)	.30
Prescription opioids	8 (3.0)	11 (2.4)	19 (2.6)	.83
Heroin	3 (1.1)	2 (0.4)	5 (0.7)	.55
Cocaine	3 (1.1)	3 (0.7)	6 (0.8)	.82
Methamphetamine	5 (1.9)	0 (0)	5 (0.7)	N/A^c^
ACE^d^, mean (SD)	2.97 (2.76)	2.67 (2.62)	2.78 (2.68)	.15

b Substance use yes/no in the last 3 months.

c N/A: not applicable.

d ACE: adverse childhood experiences.

As depicted in Table 3, an increase in the number of caregiver ACE was marginally associated with the risk of nonadherence to medication treatment and use among their children (adjusted odds ratio [OR] 0.94; *P*=.07). If a parent was the sole administrator of medications, the odds of adherence were improved (OR 1.76; *P*=.07). Relative to infants, older children had higher odds of being nonadherent. Caregivers with a higher level of education were associated with children who had lower adherence odds. Authoritative parenting style was associated with higher odds of child medication treatment and use adherence (OR 1.05; *P*=.004), while tobacco use among caregivers was associated with lower odds of child medication treatment and use adherence (OR 0.69; *P*=.04).

**Table 3.** Association between parental adverse childhood experiences (ACE) and children’s medication adherence.

Variable	Odds ratio (95% CI)		*P* value
Caregiver’s ACE	0.94 (0.88‐1.00)		.07
Who administers medication?			
Child	Reference		
Caregiver	1.76 (0.94‐3.37)		.07
Child and caregiver	0.81 (0.41‐1.62)		.55
Child’s age (y)			
<1	Reference		
1-3	0.27 (0.09‐0.82)		.02
4-6	0.46 (0.15‐1.37)		.17
7-9	0.56 (0.18‐1.70)		.31
10-12	0.56 (0.18‐1.70)		.31
13-15	0.38 (0.12‐1.22)		.11
16-18	0.37 (0.11‐1.23)		.10
Caregiver’s race			
White	Reference		
Black or African American	0.91 (0.45‐1.73)		.78
Other	0.35 (0.21‐0.58)		<.001
Caregiver’s level of education			
High school	Reference		
Some college	0.43 (0.19‐0.96)		.04
Bachelor’s degree or higher	0.46 (0.21‐1.02)		.06
Caregiver’s authoritative score	1.05 (1.01‐1.09)		.004
Caregiver’s cigarette use	0.69 (0.48‐0.98)		.04

## Discussion

This study adds to the growing population of research investigating differing types of parenting practices and factors that promote adherence to medication treatment and use. Consistent with prior research, this study showed that caregivers of children who were adherent to medication treatment and use practiced an authoritative parenting style. Uniquely, this study showed that increases in caregiver’s ACE score were only marginally associated with the risk of nonadherence to medication treatment and use.

Parents with higher ACE scores were more likely to self-report behaviors or ideas consistent with an authoritative parenting style. This could possibly be due to the fact that parents with increased levels of childhood trauma, indicated by a higher ACE score, monitor their child or children at a more optimal level, promoting a nurturing relationship. With a nurturing relationship, children may be more likely to adhere to medical recommendations and family expectations. However, a more extensive study with additional measures that could potentially impact parental ACE score, and parenting behaviors, should be conducted before assuming causation between this relationship. Excessive parental guidelines, such as those seen with an authoritarian parenting style, may deter medication treatment and use adherence levels due to the lack of a cultivating relationship. Increased parental permissiveness, such as letting their child or children decide if they would like to take their medication, could lead to decreased medication treatment and use adherence.

To combat low medication adherence, parental trauma level should be monitored in outpatient clinics, potentially through measuring ACE scores, in addition to measuring child or children trauma levels, to help provide appropriate support for both the parent and the child or children. Techniques similar to this, such as developing a screening schedule that uses age-appropriate tools to identify risk factors, are supported by the American Academy of Pediatrics [27]. Also, understanding a parent’s beliefs in regard to parental style and control may lead to fruitful discussions about behaviors that can promote medication treatment and adherence [1].

Parents with a high number of traumas may face numerous psychosocial barriers. These barriers may contribute to a status of low adherence to medication treatment and use in their child or children. Some studies have found that factors other than those included in the original ACE study, such as poverty, discrimination, or witnessing violence may hold evidentiary value in evaluating trauma and consequent adherence level [6,19,28]. It is important to take these factors and others into consideration when measuring parent and child trauma levels to ensure a well-informed treatment plan.

Even without a history of parental trauma, the provider should be prepared to educate on the reasoning for the selected treatment. By increasing a parent’s understanding of the treatment and its importance, parents may become more supportive of the prescribed regimen [1]. This would ideally lead to increased parental monitoring and subsequent increased medication adherence. In addition, this adherence could allow parents to successfully aid their child’s transition into adulthood and improve their overall trajectory.

There are limitations to this study. For instance, survey responders were predominantly Caucasian, female, employed, and earned a bachelor’s degree or higher. These demographics do not reflect the majority of the American population; therefore, it is anticipated that the generalizability of the results of this study may be limited. This study was conducted via survey, meaning it is a cross-sectional view of the population and may be subject to recall bias. The data was gathered via crowdsourcing using mTurk. A significant portion of the population who either do not have access to the internet or do not know about mTurk were unintentionally excluded, which may alter results. Of those who do use mTurk, newer accounts were prevented from participating in an effort due to their low number of prior completed tasks. Finally, while mTurk offers a new opportunity for data collection, it is up to the payer source to determine if the task completed was done appropriately. If the task is approved by the payer source, then the worker, or the parent who completed the survey in this instance, receives payment. However, if the task is declined, then the worker does not receive any compensation. In the situations where the task is denied, the worker has little ability to appeal the decision. One of the filters included a 99% approval rate in prior tasks in order to prevent fraudulent responses. A participant may have been excluded from this study if they did not have a high enough approval rating for a variety of reasons. The potential for repeated exposure to similar tasks or surveys (demonstrated by the 99% approval rate in prior tasks filter) could cause participants to think less about the tasks they were completing due to boredom, possibly leading to lower generalizability [21]. It is important to note that investigators involved in this study were not involved in the approval process by mTurk.

Future studies should explore this relationship, with a larger, more diverse study population and with a different study design to assess if these results are reproducible. Differing factors, such as parental trauma, parenting style, parental stress level, household dysfunction level, and conflict level within the home and the community, should be investigated to see whether they play a role in medication treatment and adherence collectively or individually. Trials with the intervention ideas mentioned previously should be conducted to test the effectiveness of the proposed plan to improve medication adherence. Although time cannot be reversed to address past parental traumatic exposures, this research indicates that there may be a new opportunity to start addressing child health and wellness with an intergenerational approach.

## Abbreviations

ACE: adverse childhood experiences
mTurk: Mechanical Turk
OR: odds ratio
